# Fibroblast growth factor 19 expression correlates with tumor progression and poorer prognosis of hepatocellular carcinoma

**DOI:** 10.1186/1471-2407-12-56

**Published:** 2012-02-06

**Authors:** Seiki Miura, Noboru Mitsuhashi, Hiroaki Shimizu, Fumio Kimura, Hiroyuki Yoshidome, Masayuki Otsuka, Atsushi Kato, Takashi Shida, Daiki Okamura, Masaru Miyazaki

**Affiliations:** 1 Department of General Surgery, Graduate School of Medicine, Chiba University, 1-8-1, Inohana, Chuo-ku, Chiba 260-0856, Japan

## Abstract

**Background:**

Although fibroblast growth factor 19 (FGF19) can promote liver carcinogenesis in mice, its involvement in human hepatocellular carcinoma (HCC) has not been well investigated. FGF19, a member of the FGF family, has unique specificity for its receptor FGFR4. This study aimed to clarify the involvement of FGF19 in the development of HCC.

**Methods:**

We investigated human FGF19 and FGFR4 expression in 40 hepatocellular carcinoma specimens using quantitative real-time reverse transcription polymerase chain reaction (RT-PCR) analysis and immunohistochemistry. Moreover, we examined the expression and the distribution of FGF19 and FGFR4 in 5 hepatocellular carcinoma cell lines (HepG2, HuH7, HLE, HLF, and JHH7) using RT-PCR and immunohistochemistry. To test the role of the FGF19/FGFR4 system in tumor progression, we used recombinant FGF19 protein and small interfering RNA (siRNA) of *FGF19 *and *FGFR4 *to regulate their concentrations.

**Results:**

We found that FGF19 was significantly overexpressed in HCCs as compared with corresponding noncancerous liver tissue (*P *< 0.05). Univariate and multivariate analyses revealed that the tumor *FGF19 *mRNA expression was an independent prognostic factor for overall and disease-free survival. Moreover, we found that the FGF19 recombinant protein could increase the proliferation (*P *< 0.01, *n *= 12) and invasion (*P *< 0.01, *n *= 6) capabilities of human hepatocellular carcinoma cell lines and inhibited their apoptosis (*P *< 0.01, *n *= 12). Inversely, decreasing *FGF19 *and *FGFR4 *expression by siRNA significantly inhibited proliferation and increased apoptosis in JHH7 cells (*P *< 0.01, *n *= 12). The postoperative serum FGF19 levels in HCC patients was significantly lower than the preoperative levels (*P *< 0.01, *n *= 29).

**Conclusions:**

FGF19 is critically involved in the development of HCCs. Targeting FGF19 inhibition is an attractive potential therapeutic strategy for HCC.

## Background

Hepatocellular carcinoma (HCC) is a highly aggressive solid tumor associated with poor prognosis [1]. Curative therapies of surgical treatment, including hepatic resection and liver transplantation, improve the chances of survival of patients with HCC [2-4]. However, a limited number of patients can be treated with surgery because of the damage to liver function. The prognosis for most patients remains poor after surgery for multicentric recurrence and intrahepatic metastasis [5,6]. Therefore, the development of a systemic therapy that targets a new molecule involved in HCC is needed.

Fibroblast growth factor (FGF) signaling plays an important role in a variety of processes, including proliferation, cellular differentiation, wound repair, and angiogenesis [7-9]. It has been reported that amplification or overexpression of FGFs is associated with the pathogenesis of malignant neoplasms, such as leukemias and sarcomas as well as stomach, pancreas, bladder, colon, breast, and prostate cancer [10-15]. Further, several correlations between overexpression, polymorphism, translocation, and truncation of FGF receptor (FGFR) and a variety of human neoplasms such as myeloma, breast, stomach, colon, bladder, and cervical cancer have also been reported [16-21]. Therefore, the FGF/FGFR system plays a critical role in tumor progression.

FGF19 was first identified on the basis of its amino acid similarities to murine FGF15 (53% identity) [22]. FGF19 is currently unique in displaying apparent specificity for FGFR4 [23,24]. Previous studies have reported that FGF19 and hepatocyte FGFR4 regulate biosynthesis in the bile duct by repression of *CYP7A1 *[25]. Further, FGF19 reportedly increases the metabolic rate, reduces body weight, and reverses diabetes in both high-fat-fed mice and leptin-deficient mice [26]. On the other hand, ectopic expression of FGF19 in mice promotes hepatocyte proliferation, hepatocellular dysplasia, and neoplasia [27]. Moreover, recent reports have revealed that a neutralizing antibody that selectively blocks the interaction of FGF19 with FGFR4 inhibits the growth of colon tumors and the formation of liver tumors *in vivo *[28]. However, although the involvement of FGF19 in HCC has been demonstrated, no study has so far addressed the significance of FGF19 expression or clarified its role in the mechanism of HCC development in humans [29].

In this study, we hypothesized that the FGF19/FGFR4 system is activated in patients with HCC and is correlated with the aggressiveness of the tumor. We elucidated the association between the FGF19/FGFR4 system and the development of HCC using human samples and *in vitro *experimental models.

## Methods

### Patients and specimens

Cancerous tissues and surrounding non-cancerous hepatic parenchyma were obtained from 40 primary HCC Japanese patients who underwent curative resection surgery at Chiba University Hospital, Japan, from January 1995 to December 2001. They did not have any other malignancies. The ethics committee of Chiba University Hospital approved this study. Informed consent was obtained from every patient for the use of resected tissue before the study began. Samples were obtained from 31 men and 9 women aged 49-78 years. Specimens were histologically classified by the Japanese staging system of the Liver Cancer Study Group of Japan [30]. In the corresponding noncancerous parenchyma, cirrhosis was found in 14 patients (35%).

### Cells

The human HCC cell lines HuH7, HepG2, HLE, HLF, PLC/PRF/5, JHH1, JHH2, JHH5, JHH6, and JHH7 were obtained from the Health Science Research Resources Bank (Osaka, Japan). HuH7, HepG2, HLE, HLF, and PLC/PRF/5 were cultured in Dulbecco's Modified Eagle's Medium containing 10% fetal bovine serum (Sigma-Aldrich Corp., St. Louis, MO). JHH1, JHH2, JHH5, JHH6, and JHH7 were cultured in William's Medium E with 10% fetal bovine serum. Primary cultures of human hepatocytes were prepared and cultured as described in previous papers [31].

### RNA extraction and cDNA synthesis

Total RNA from the cell lines and tissues was isolated using RNeasy Mini Kits (Qiagen, Hilden, Germany) according to the manufacturer's protocol. Specimens were taken from viable and non-fibrotic areas of tumors and noncancerous tissues. They were quickly frozen in liquid nitrogen and then stored at -80°C until use. Synthesis of cDNA from total RNA was conducted using a Ready-to-Go^® ^cDNA synthesis kit (Amersham Biosciences Corp., Piscataway, NJ) following the manufacturer's protocols.

### Real-time quantitative RT-PCR analysis

The expressions of *FGF19 *and *FGFR4 *were examined by reverse transcription polymerase chain reaction (RT-PCR) with the following primers: (forward) (5'-TCT CCT CTG ACT TCA ACA GCG ACA C-3') and (reverse) (5'-TGT TGC TGT AGC CAA ATT CGT TGT C-3') for human GAPDH, (forward) (5'-CAG CTG TAC AAG AAC AGA GGC TTT C-3') and (reverse) (5'-AAA TGG GTC CAT GCT GTC GGT CTC C-3') for FGF19, and (forward) (5'-CAT CCG CTG GCT TAA GGA TGG AC-3') and (reverse) (5'-ATC ACG AGA CTC CAG TGC TGA TG-3') for FGFR4. All PCR reactions were performed using the SYBR Green PCR Core Reagents kit (Perkin-Elmer Applied Biosystems, Foster City, CA, USA) under the following conditions: 1 cycle at 95°C for 10 min, 50 cycles at 95°C for 10 sec, 60°C for 5 sec, 72°C for 10 sec, and 80°C for 1 sec. Real-time detection of the SYBR Green emission intensity was conducted with a LightCycler^® ^(Roche, Mannheim, Germany). An equivalent amount of cDNA sample, derived from 40 ng total RNA, was used for each PCR reaction. The mRNA in each sample was then automatically quantified with reference to the standard curve constructed with each use of the LightCycler^®^. Quantitative RT-PCR was performed at least three times per sample. To standardize the amount of RNA, we quantified the expression of *GAPDH *mRNA in each sample and then divided the amounts of expressed *FGF19 *and *FGFR4 *mRNA by that of *GAPDH*.

### Immunohistochemical staining

An immunohistochemical analysis was performed on paraffin-embedded sections using the Envision kit (Dako, Glostrup, Denmark) following the manufacturer's instructions. The sections were boiled in retrieval solution to expose antigens. Anti-FGF19 (R&D Systems, Inc., MN) monoclonal antibodies, anti-FGFR4 (Santa Cruz Biotechnology, Inc., Santa Cruz, CA) monoclonal antibodies and control antibody (R&D Systems, Inc., MN) were applied as the primary antibodies to the sections at a dilution of 25 μg/mL and 1:50. The section slides were counterstained with hematoxylin, dehydrated, and mounted. The immunostaining was evaluated independently by two pathologists.

### Cell fixation and staining

HuH7, HepG2, HLE, HLF, and JHH7 cells (1 × 10^4^/well) were seeded and cultured at 37°C in a humidified 5% CO_2 _atmosphere for 24 h. Then, the cells were washed 3 times with phosphate-buffered saline (PBS) and fixed in 100% acetone for 10 min at 4°C. After fixation, cells were washed 3 times with PBS. Cells were blocked in blocking serum at room temperature for 1 h in a humidified chamber. Cells were washed 3 times with PBS and stained at the same dilution as paraffin-embedded sections.

### Cell proliferation assay with FGF19 recombinant protein

Cells were seeded (6 × 10^3^/well) with FGF19 recombinant protein (Cell Sciences, MA, USA) at concentrations of 0.1, 1, 10, or 100 ng/mL and cultured. After 48, 72, or 96 h, 20 μL of Cell Titer 96^® ^AQ_ueous _(Promega, WI, USA) was added to the culture media then incubated for 1 h at 37°C in a humidified 5% CO_2 _atmosphere. Then, the absorbance of each of the plates at 490 nm was recorded using a 96-well plate reader (Bio-Rad, CA, USA). The proliferation index (PI) was defined as OD values of the recombinant protein-treated cells divided by those of the untreated control cells. We examined proliferation assay with 12 chambers in the same condition at the same time.

### Cell apoptosis assay with FGF19 recombinant protein

Cells were harvested at 48 h after seeding and incubation with 1 ng/ml of FGF19 recombinant protein. To initiate apoptosis, 5-fluorouracil (5-FU) (Roche) was added at a final concentration of 10 μg/mL. After further incubation and gravity sedimentation for 24 h, the supernatant was removed carefully and the cell pellets were resuspended in 200 μL lysis buffer. Then, ELISA was performed using the Cell Death Detection Kit (Roche) according to the manufacturer's specifications. The apoptosis index (AI) was defined as OD values of recombinant protein-treated cells divided by those of the controls. We examined apoptosis assay with 12 chambers in the same condition at the same time.

### Invasion assay with FGF19 recombinant protein

Double-chamber transwell plates with 8-μm pore-size polycarbonate membrane inserts at the base of the upper chamber (Cell Biolabs, Inc., San Diego, CA) were used. Here, 300 μL of warm, serum-free media was added to each upper chamber and incubated for 1 h at 37°C. Upper chambers containing the reconstituted Matrigel membranes were set into the lower chambers of the 24-well cluster plates. JHH7 cells were trypsinized, counted, and resuspended with fresh medium before being plated into the upper chambers of the assay wells. Approximately 1 × 10^6 ^cells were plated in 300 μL of defined medium into each upper chamber. Then, 500 μL of medium was added to each lower chamber. Both the media in the upper and lower chambers contained 1 ng/ml of human recombinant FGF19. JHH7 were cultured in this way for 48 h in a humidified 5% CO_2 _atmosphere. The medium in the upper chamber was then aspirated, and cells from its inner surface were wiped off using a cotton swab. The membranes were stained with 400 μL of Cell Stain Solution and incubated for 10 min at room temperature. Stained inserts were gently washed several times with water. Then, the stained inserts were transferred to an empty well containing 200 μL of extraction solution and incubated for 10 min on an orbital shaker. Subsequently, 100 μL from each sample was transferred to a 96-well microtiter plate, and the OD 560 nm of each sample was measured in a 96-well plate reader (Bio-Rad, Hercules, CA). We examined invasion assay with 6 chambers in the same condition at the same time.

### Migration assay with FGF19 recombinant protein

Migration assay was performed by using transwell plates with 8 μm pore (BD Biosciences). JHH7 cells (1 × 10^5^) suspended in 500 μl serum-free Williams medium were seeded into the upper part, whereas the lower compartment was filled with 1 ml medium with 1 ng/ml of the human FGF19 recombinant protein. After incubation for 24 h at 37°C in 5% CO_2_, non-migrating cells were removed from the upper surface of the membrane by scrub. Cells on the reverse side were stained with 0.1% crystal violet, and counted under a microscope at x100 magnification. We examined migration assay with 6 chambers in the same condition at the same time.

### Gene knockdown using small interfering RNA transfection

Transfections of *FGF19, FGFR4 *and non-targeting negative control small interfering RNA (siRNA) (AMBION, Austin, TX) were conducted with Lipofectamine 2000 (Invitrogen, Carlsbad, CA) in 96-well plates according to the manufacturer's specifications. The day before transfection, the JHH7 cells were trypsinized, counted, and seeded at 6 × 10^3 ^cells per well into 96-well plates. Lipofectamine 2000 diluted in Opti-MEM (Invitrogen) was supplemented to the siRNA mixture. The mixture was incubated for 20 min at room temperature. The mixture and Opti-MEM were added to the plate to a final siRNA concentration of 20 nM.

### Flow cytometry

Control and *FGFR4*-specific siRNA were transfected into JHH7 as described above. At 72 h after transfection, the cells were collected and stained with either a phycoerythrin-conjugated anti-FGFR4 antibody (BioLegend, San Diego, CA) or an isotype-matched control (R&D systems, Minneapolis, MN). Flow cytometric analysis of cell surface FGFR4 was performed using a FACSCalibur analyzer (Becton Dickinson Immunocytometry Systems, San Jose, CA).

### Cell proliferation assay after FGF19 and FGFR4 siRNA transfection

Cells (6 × 10^3 ^per well) were seeded into 96-well plates, cultured for 24 h, and transfected with control, *FGF19*, or *FGFR4 *siRNA. At 72 h after transfection, cells were harvested. Then, 20 μL of Cell Titer 96^® ^AQ_ueous _(Promega) was added to the culture media and incubated for 2 h at 37°C in a humidified 5% CO_2 _atmosphere. The absorbance of the plates at 490 nm was recorded using a 96-well plate reader (Bio-Rad).

### Apoptosis assay after FGF19 and FGFR4 siRNA transfection

Cells (6 × 10^3 ^per well) were seeded into 96-well plates, cultured for 24 h, and transfected with control, *FGF19*, or *FGFR4 *siRNA. Cells were harvested at 72 h after transfection. To initiate apoptosis, 5-FU (Roche) at a final concentration of 10 μg/mL was added. After further incubation and gravity sedimentation for 24 h, the supernatant was carefully removed, and the cell pellets were resuspended in 200 μL lysis buffer. Then, ELISA was performed using the Cell Death Detection Kit (Cell Biolabs, Inc.) according to the manufacturer's specifications.

### Changes in serum FGF19 levels in patients with HCC after hepatectomy

Blood serum was obtained from 10 healthy subject and 29 primary HCC patients who underwent curative resection at Chiba University Hospital, Japan, from 2005 to 2007 (22 men, 7 women). The samples were obtained on the day before and the day after surgery. A sandwich ELISA kit was used for colorimetric detection of FGF19 in serum (FGF19 Quantikine ELISA kit, Minneapolis, MN) following the manufacturer's instructions.

### Statistical analysis

The relative mRNA expression levels (*FGF19/GAPDH *and *FGFR4/GAPDH*) were calculated from the quantified data. Mann-Whitney's *U *test was used to analyze the differences in the *FGF19 *and *FGFR4 *expression levels between HCCs and the corresponding noncancerous hepatic tissues. To analyze the correlation between *FGF19, FGFR4*, and clinicopathological parameters, differences in the numerical data between the two groups were evaluated using the Kruskal-Wallis test. Overall and disease-free survival rates were then calculated using the Kaplan-Meier method, and the differences in survival curves were analyzed using the log-rank test. Survival was counted if the patient was still alive or had died of other causes. Independent prognostic factors were analyzed by the Cox proportional hazards regression model in a stepwise manner. All the statistical analyses were performed using Stat View software (Version 5.0, Abacus Concepts, Berkeley, CA). Data are expressed as mean ± SE. *P *< 0.05 denoted the presence of a statistically significant difference.

## Results

### FGF19 and FGFR4 expression in HCC

**Figure 1:**
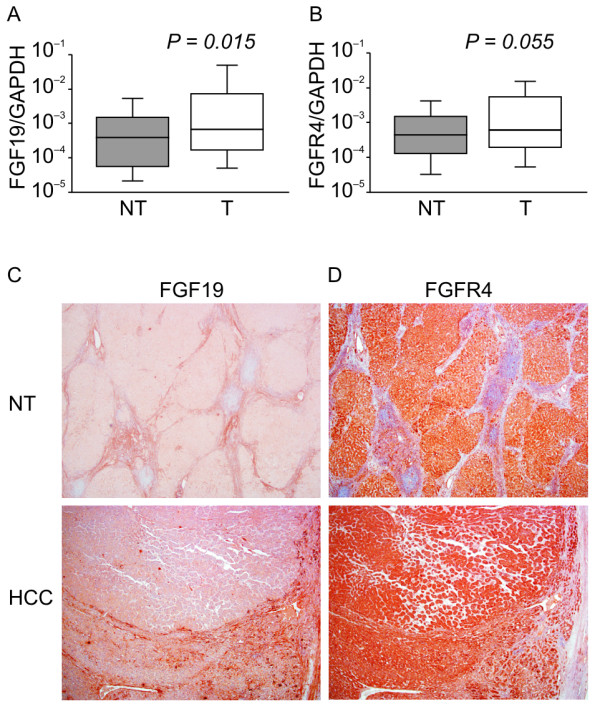
**Real-time quantitative RT-PCR analysis, immunohistochemical staining of representative specimens from HCC patients of 40 HCC samples**. (**A**): Ratio of average *FGF19/GAPDH *expression in HCC (T) compared with corresponding noncancerous hepatic tissues (N). The average *FGF19/GAPDH *level in HCCs. (**B**): The average *FGFR4/GAPDH *level in HCCs. (**C**): Immunohistochemistry using anti-FGF19 monoclonal antibodies; HCC tissue (lower) and noncancerous hepatocytes (upper). (**D**): Immunohistochemistry using anti-FGFR4 monoclonal antibodies; HCC tissue (lower) and noncancerous tissue (upper). (Original magnifications: ×40 (upper); ×40 (lower)). RT-PCR; reverse transcription polymerase chain reaction; HCC, hepatocellular carcinoma.

We examined 40 HCC samples and corresponding noncancerous hepatic tissues for *FGF19 *mRNA expression using real-time quantitative RT-PCR. The average *FGF19/GAPDH *level in HCCs was significantly higher than that in noncancerous tissues. (Figure 1A; *P *= 0.015), whereas *FGFR4 *was not significantly overexpressed in HCCs compared to noncancerous tissues (Figure 1B, *P *= 0.055). An immunohistochemical study using anti-FGF19 monoclonal antibodies was performed to determine whether FGF19 protein was expressed in HCC specimens. FGF19 protein was detectable in both cancer and noncancerous tissues Figure 1C). FGF19 staining was observed in the cytoplasm of tumor cells and noncancerous hepatocytes, and tended to be greater in the tumor cells. FGFR4 staining was not significantly greater in the membranes of tumor cells as compared to those of cells from corresponding noncancerous tissue (Figure 1D).

### Correlation between FGF19 mRNA expression and clinicopathological parameters in HCC

The correlation between *FGF19 *expression and clinicopathological parameters in patients with HCC is shown in Table 1. Significant correlation was found only between *FGF19 *mRNA expression and pathological stage. (Table 1, *P *= 0.049).

**Table 1:** Correlations between *FGF19 *expression and clinicopathological parameters in 40 patients with HCC

Clinicopathological parameters	Patient number	Tumor *FGF19 *mRNA* (Mean ± SE)	*P *value
Age			
< 65 years	23	0.007 ± 0.006	0.505
≥65 years	17	0.016 ± 0.014	
Gender			
Male	31	0.014 ± 0.009	0.319
Female	9	0.003 ± 0.002	
Virus			
HBV	4	0.058 ± 0.058	0.808
HCV	27	0.002 ± 0.001	
None	9	0.017 ± 0.016	
Miran Criteria			
In	17	0.001 ± 0.000	0.194
Out	23	0.0019 ± 0.011	
AFP			
< 10 ng/mL	20	0.013 ± 0.012	0.194
≥ 10 ng/mL	20	0.009 ± 0.007	
Tumor size			
< 5 cm	20	0.001 ± 0.000	0.137
≥5 cm	20	0.021 ± 0.013	
Histologic type of tumor			
Well differentiated	6	0.001 ± 0.001	0.808
Moderately differentiated	31	0.014 ± 0.009	
Poorly differentiated	3	0.002 ± 0.001	
Formation of fibrous capsule			
Present	39	0.011 ± 0.007	-------
Absent	1	0.0002	
Capsular infiltration			
Present	38	0.012 ± 0.007	0.718
Absent	2	0.0003 ± 0.0003	
Septal formation			
Present	30	0.014 ± 0.009	0.445
Absent	10	0.002 ± 0.002	
Serosal invasion			
Present	1	0.016	-------
Absent	39	0.011 ± 0.007	
Portal invasion			
Present	30	0.014 ± 0.009	0.449
Absent	10	0.002 ± 0.002	
Hepatic vein invasion			
Present	10	0.024 ± 0.023	0.269
Absent	30	0.007 ± 0.005	
Intrahepatic metastasis			0.580
Present	22	0.008 ± 0.006	
Absent	18	0.015 ± 0.013	
Bile duct invasion			
Present	1	0.232	-------
Absent	39	0.005 ± 0.004	
Lymph node metastasis			
Present	1	0.001	-------
Absent	39	0.011 ± 0.007	
Pathological stage			
II	7	0.0004 ± 0.0002	0.049
III	14	0.020 ± 0.016	
IV	19	0.009 ± 0.007	

*HBV *hepatitis B virus, *HCV *hepatitis C virus, *AFP *alpha-fetoprotein, *S.E*. standard error

### Univariate and multivariate analysis of prognostic factors for patients with HCC

**Figure 2:**
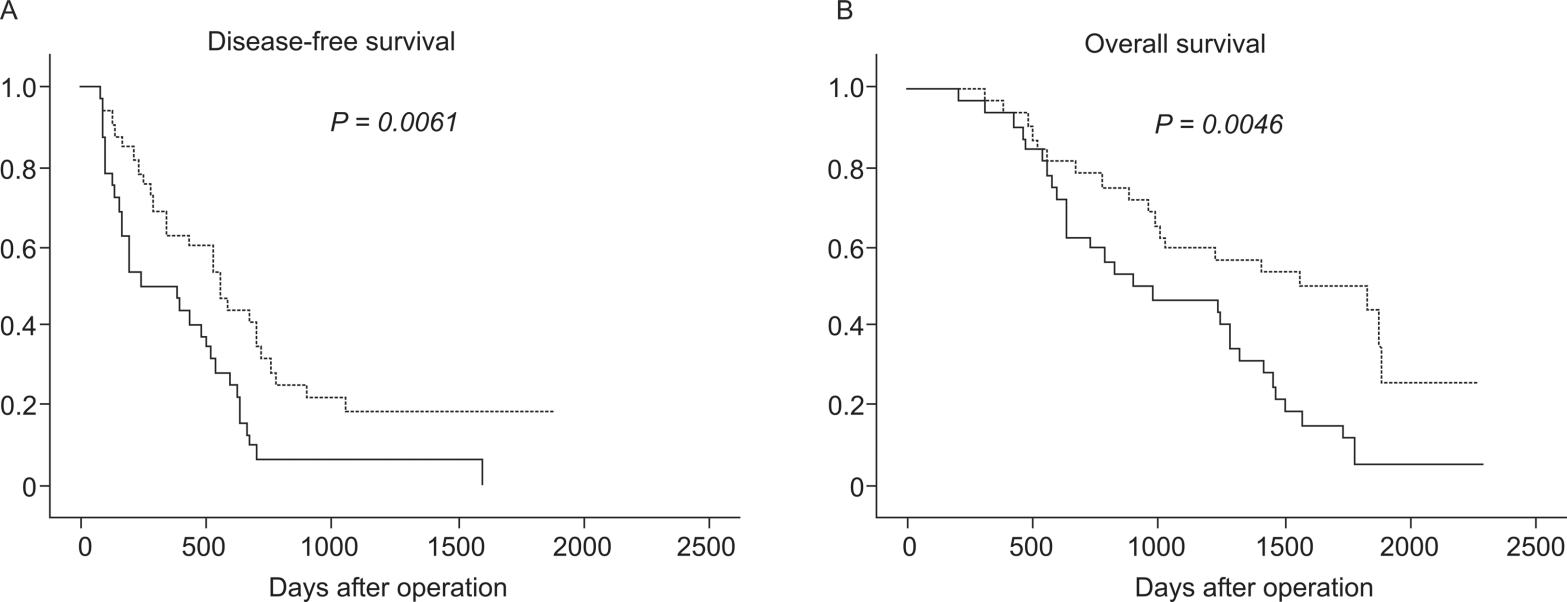
**Survival rates of 40 patients with HCC**. (**A**) Disease-free survival rates of patients with HCCs. (**B**) Overall survival rates of patients with HCCs. High expression of *FGF19 *mRNA was significantly associated with poorer prognosis.

For statistical analysis of *FGF19 *levels, the specimens were divided into two groups based on the median value of tumor *FGF19 *mRNA (5.7 × 10^-4^): a high expression group (n = 20) and a low expression group (n = 20). We analyzed disease-free survival rates and overall survival rates to assess the prognostic significance of *FGF19*. The 5-year disease-free and overall survival rates of the 40 patients with HCC were 10% and 40%, respectively. Using the Kaplan-Meier curve assessment, we found that patients with higher FGF19 expression levels had lower 5-year survival rates than patients with lower FGF19 expression levels (disease-free survival rate, 0% vs. 20%; *P *= 0.0061; overall survival rate, 20% vs. 60%; *P *= 0.0046; as shown in Figure 2). To evaluate the potential of using FGF19 expression in determining the postoperative prognosis of HCC patients, univariate analysis using a Cox proportional hazard regression model was conducted (Table 2). The results showed that the serum AFP index, *FGF19 *mRNA level, and intrahepatic metastasis were significant predictors of disease-free survival (*P *= 0.004, 0.021, and 0.020, respectively), while Milan criteria, serum AFP index, *FGF19 *mRNA level, tumor size, and intrahepatic metastasis were significant predictors of overall survival (*P *= 0.006, 0.042, 0.018, 0.028 and 0.020, respectively). Multivariate analysis was conducted with 5 prime variables that are regarded as prognostic factors for patients with HCC in previous studies because of the small sample size of patients (Table 3)[30]. A high *FGF19 *mRNA level remained significant in both disease-free survival and overall survival, and was the strongest in overall survival (*P *= 0.040 and 0.003, odds ratio = 2.34 and 3.61, respectively).

**Table 2:** Univariate analysis of disease-free and overall patient survivals with hepatocellular carcinoma

Clinicopathological Parameters	Median Disease-free survival	*P *value	Median Overall survival	*P *value
Age				
< 65 years	237	0.349	1267	0.673
≥ 65 years	594		1234	
Gender				
Male	673	0.079	1283	0.568
Female	283		1092	
Virus				
HBV	237	0.178	1211	0.968
HCV	325		1283	
None	847		1234	
Miran Criteria				
In	847	0.061	1940	0.006
Out	256		572	
AFP				
High (n = 20)	237	0.004	573	0.042
> 10 ng/mL				
Low (n = 20)	847		1818	
≤ 10 ng/mL				
FGF19T				
High (n = 20)	283	0.021	628	0.018
Low (n = 20)	413		1758	
Tumor size				
< 5 cm	717	0.171	1428	0.028
≥ 5 cm	256		498	
Histologic type of tumor				
Well differentiated	673	0.813	1428	0.712
Moderately differentiated	323		1234	
Poorly differentiated	594		1092	
Formation of fibrous capsule				
Present	413	-------	1267	-------
Absent	64		64	
Capsular infiltration				
Present	403	0.853	1234	0.725
Absent	64		64	
Septal formation				
Present	325	0.872	1230	0.777
Absent	413		1283	
Serosal invasion				
Present	104	-------	172	-------
Absent	413		1267	
Portal invasion				
Present	283		825	0.105
Absent	881		1940	
Hepatic vein invasion				
Present	256	0.215	573	0.150
Absent	597		1428	
Intrahepatic metastasis				
Present	283	0.020	628	0.020
Absent	881		1983	
Bile duct invasion				
Present	403	-------	1267	-------
Absent	413		573	
Lymph node metastasis				
Present	256	-------	347	-------
Absent	413		1267	
Pathological stage				
II	1373	-------	2085	0.060
III	594		1234	
IV	237		572	

*HBV *hepatitis B virus, *HCV *hepatitis C virus, *AFP *alpha-fetoprotein

**Table 3:** Multivariate analysis of disease-free and overall patient survivals with hepatocellular carcinoma

Variable	Disease-free survival			Overall survival		
	
	Hazard Ratio	**95% C.I**.	*P *value	Hazard Ratio	**95% C.I**.	*P *value
Portal invasion (0-4)	2.59	1.27-5.28	0.009	2.74	1.31-5.73	0.007
AFP	2.49	1.09-5.72	0.031	2.03	0.84-4.91	0.114
(≤ 10 ng/mL: > 10 ng/mL)						
Tumor size	0.58	0.14-2.35	0.445	0.87	0.21-3.56	0.845
(≤ 5 cm: > 5 cm)						
Intrahepatic metastasis	1.25	0.60-2.60	0.550	1.53	0.66-3.53	0.319
(absent: present)						
Miran criteria	0.31	0.76-1.27	0.105	0.28	0.07-1.19	0.085
(in: out)						
tumor FGF19	2.34	1.04-5.26	0.040	3.61	1.56-8.36	0.003
(high: low)						

*AFP *alpha-fetoprotein, *C.I*. confidence interval

### FGF19 expression of the HCC cell lines

**Figure 3:**
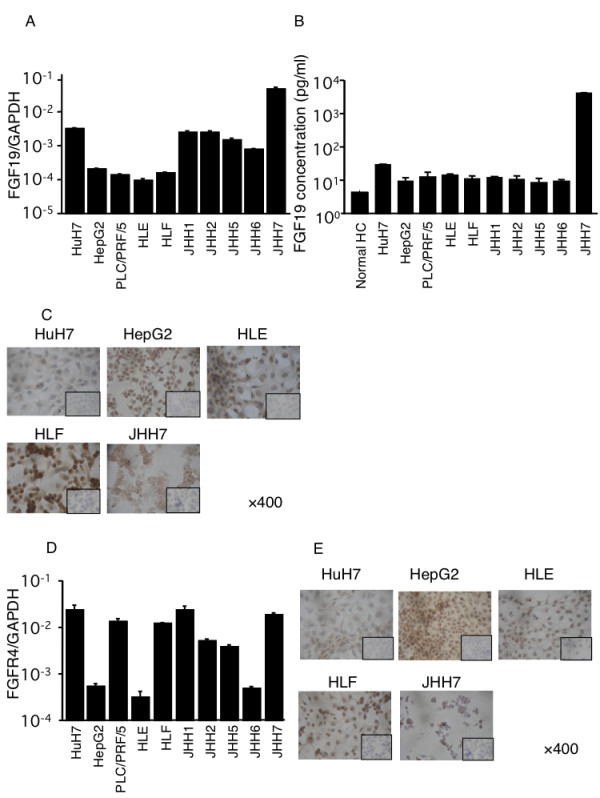
**(A) *FGF19 *mRNA expression in cell extracts from HuH7, HepG2, PLC/PRF/5, HLE, HLF, JHH1, JHH2, JHH5, JHH6, and JHH7 cell lines was examined using quantitative RT-PCR (*n *= 8)**. *FGF19 *was expressed in all cell lines. (**B**) FGF19 protein levels in the supernatant media from ten cell lines and normal hepatocytes (normal HC) were assayed by ELISA. FGF19 was detected in the supernatant of all of them. (**C**) Using anti-FGF19 monoclonal antibodies, diffuse positive staining was demonstrated in the cytoplasm of HuH7, HepG2, HLE, HLF, and JHH7 cells. The closed rectangles indicate the same cells stained Immunohistochemically using control antibody. (**D**) *FGFR4 *mRNA expression in cell extracts from HuH7, HepG2, PLC/PRF/5, HLE, HLF, JHH1, JHH2, JHH5, JHH6, and JHH7 cell lines was examined using quantitative RT-PCR (*n *= 8). *FGFR4 *was expressed in all cell lines. (**E**) Using anti-FGFR4 monoclonal antibodies, positive staining was demonstrated in the cell membranes of all of the above HCC lines. (Original magnifications (**C**) × 400), (**E**) × 400)) The closed rectangles indicate the same cells stained Immunohistochemically using control antibody.

We used RT-PCR to analyze the differential expression of *FGF19 *by measuring mRNA levels among 10 HCC lines. *FGF19 *mRNA was expressed in all HCC lines, and at a remarkably higher level in JHH7 (Figure 3A). FGF19 protein was detected using an FGF19 ELISA kit in culture media for all human HCC lines and normal hepatocyte at 72 h after seeding; FGF19 was noted to be significantly higher in JHH7 cells and significantly lower in normal hepatocyte (Figure 3B). An immunocytohistochemical study using anti-FGF19 monoclonal antibodies was performed to determine whether FGF19 is expressed in all HCC lines; FGF19 protein was detectable in all HCC lines in the cytoplasm (Figure 3C). Similarly, *FGFR4 *mRNA was expressed in all HCC lines (Figure 3D). Through FGFR4 staining, FGFR4 protein was observed in all HCC lines (Figure 3E).

### Proliferation, apoptosis assay, invasion, and migration assay with FGF19 recombinant protein

**Figure 4:**
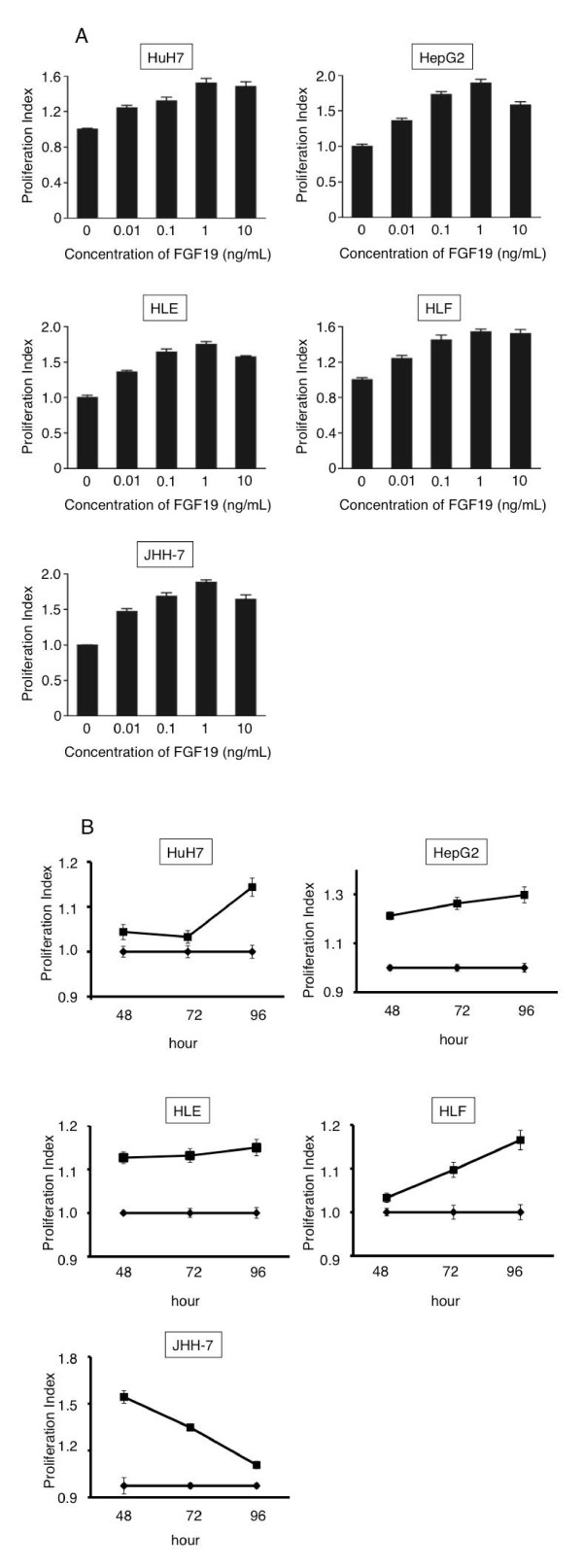
**(A) Proliferation changes after addition of various concentration of the FGF19 recombinant protein**. (**B**) The proliferation index (PI) was defined as the OD values of cells treated with recombinant protein divided by those of untreated cells. The PI increased after addition of FGF19 recombinant protein (0.01-10 ng/mL final concentration) to culture media and culturing for 96 h. PI, proliferation index.

**Figure 5:**
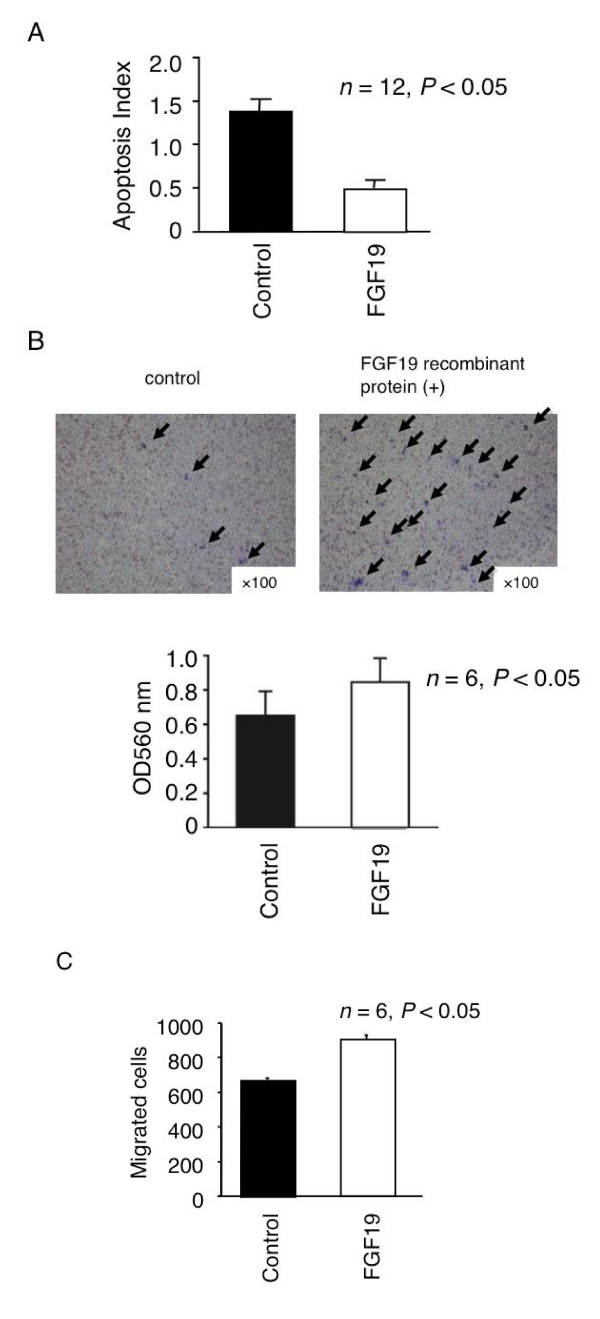
**(A) Apoptosis assay at 72 h after FGF19 recombinant protein treatment**. (left) 5-FU treatment; (right) 5-FU and recombinant protein treatment. The apoptosis index (AI) was defined as the OD values of cells treated with these agents divided by those of untreated cells. (**B**) (upper) Invasion assay after FGF19 recombinant protein treatment (100× magnification). (lower) Invasion assay 48 h after FGF19 recombinant protein treatment after FGF19 recombinant protein treatment. (**C**) FGF19 enhanced cell migration ability in JHH7 cell line. The transwell system was used to evaluate migratory ability. Migrated cells were counted and the quantitative results are shown (n = 6). The data presented are from a representative experiment, being quantitatively similar in the replicate experiments.

We chose the HuH7, HepG2, HLE, HLF, and JHH7 cell lines upon consideration of the combination of FGF19 and FGFR4 levels. We cultured these HCC lines with FGF19 recombinant protein at concentrations of 0.01, 0.1, 0.5, 1, 5, 10, 50, or 100 ng/mL and performed a proliferation assay (Figure 4A; *n *= 12, *P *< 0.05). We found that the proliferation of all HCC cells examined increased significantly upon addition of FGF19 recombinant protein at concentrations of 0.01-10 ng/mL over 48-96 h (Figure 4B). The proliferation index was highest when the concentration of FGF19 was 1 ng/mL in the 5 HCC lines, whereas apoptosis of HCC cells was significantly suppressed by addition of 1 ng/mL FGF19 recombinant protein in culture media (Figure 5A; *n *= 12, *P *< 0.05). Tumor metastasis is still a major problem in management of cancer. In order to investigate further whether FGF19 plays an important role in tumor metastasis, the invasion assay and migration assay were performed. Invasion assays were used to investigate the alteration of cancer cell invasiveness in the presence of FGF19 recombinant protein for JHH7. The assays were conducted by staining and measuring the OD at 560 nm; they revealed that 1 ng/mL of the FGF19 recombinant protein significantly increased the mobility and invasiveness of the JHH7 cells (Figure 5B; *n *= 6, *P *< 0.05). As shown in Figure 5C, the number of migrated cells also increased dramatically after addition of the FGF19 recombinant protein (Figure 5C; *n *= 6, *P *< 0.05). Results of the invasion assay and migration assay indicate that FGF19 may be an activator of tumor metastasis.

### Proliferation and apoptosis assay after FGF19 and FGFR4 siRNA

**Figure 6:**
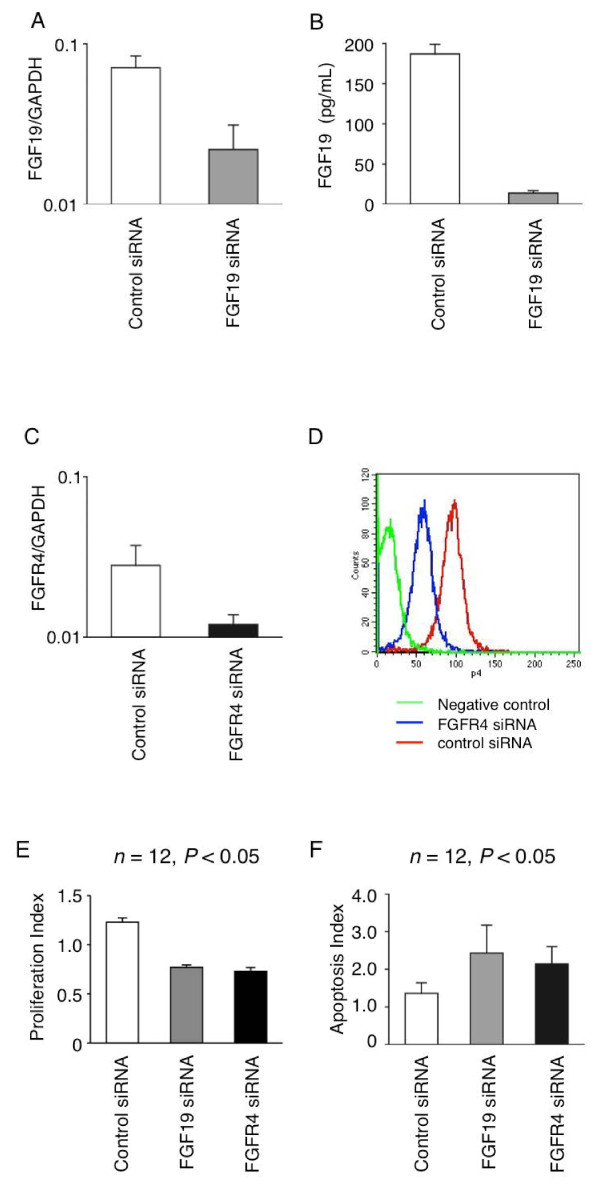
**Suppression of FGF19 expression was confirmed at transcript level by RT-PCR**. **(A) **and at protein level by ELISA analysis **(B)**. Analysis was performed at 48 h and 72 h following siRNA transfection for mRNA and protein level, respectively. *FGF19 *mRNA expression was suppressed to 30% of control siRNA in *FGF19*-specific siRNA-treated cells. n = 12, P < 0.05 at top of the Figure 6A, 6B, and 6C (like figure 6E and 6D). *FGFR4 *gene silencing by siRNA. Suppression of *FGFR4 *expression was confirmed at transcript level by RT-PCR (**C**) and at protein level by flow cytometry (**D**). Analysis was performed at 48 h and 72 h following siRNA transfection for mRNA and protein levels, respectively. *FGFR4 *mRNA expression was suppressed to 40% of control siRNA in *FGFR4*-specific siRNA-treated cells. n = 12, P < 0.05 at top of the Figure 6A, 6B, and 6C (like figure 6E and 6D). (**E**) Proliferation assay in JHH7 cells at 72 h after *FGF19/FGFR4 *siRNA treatment. (**F**) Apoptosis assay in JHH7 cells at 96 h after *FGF19/FGFR4 *siRNA treatment. PI, Proliferation Index. AI, Apoptosis Index.

We selected the JHH7 cell line for further studies because this HCC line showed the highest *FGF19 *expression at both the mRNA and protein levels in the 10 HCC lines examined. Transfection of *FGF19 *siRNA decreased *FGF19 *mRNA expression by 72% after 48 h as compared with cells transfected with control siRNA (Figure 6A). Maximum downregulation of FGF19 protein occurred at 72 h after transfection. The FGF19 protein was downregulated by more than 90% in the JHH7 cells (Figure 6B). Similarly, *FGFR4 *siRNA transfection downregulated mRNA (Figure 6C) and protein expression of FGFR4 (Figure 6D). Further, proliferation of JHH7 cells was significantly suppressed by *FGF19 *siRNA transfection (Figure 6E; *n *= 12, *P *< 0.05) and *FGFR4 *siRNA transfection (*n *= 12, *P *< 0.05). We also found that apoptosis induced by 5-FU increased significantly in the *FGF19 *siRNA group at 96 h after transfection (Figure 6F; *n *= 12, *P *< 0.05) and *FGFR4 *siRNA transfection (*n *= 12, *P *< 0.05).

### Changes in serum FGF19 level in patients with HCC after hepatectomy

**Figure 7:**
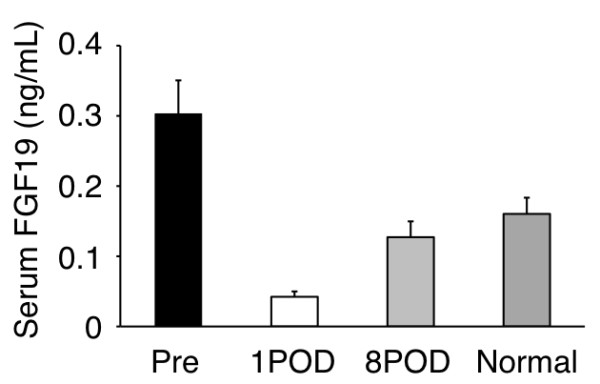
**The changes in serum FGF19 levels, as measured by ELISA, of the 29 patients with HCCs from 1^st ^day and the 8^th ^day after curative resection surgery**. Normal shows serum FGF19 levels of the 10 health subjects.

Serum FGF19 levels were measured at the pre- and postoperative period. Serum FGF19 levels of 10 normal subject were also measured. Postoperative FGF19 levels were significantly lower than those in the preoperative period (Figure 7; *n *= 29, *P *< 0.05). Moreover, FGF19 levels in the normal subjects were significantly lower than those in the preoperative period and significantly higher than those in the postoperative period (Figure 7, *P *< 0.05).

## Discussion

In this study, we clarified the association between the FGF19/FGFR4 system and the development of HCC using human samples and *in vitro *experimental models. *FGF19 *was expressed at significantly higher levels in HCCs, whereas the receptor *FGFR4 *was expressed in noncancerous tissue and HCC at similar levels. Han Kiat Ho et al., reported that one third of HCC patients exhibited increased FGFR4 mRNA expression in the matched tumor/normal tissue [29]. In our study, FGFR4 mRNA expression showed no significant difference but increased tendency (Figure 1B, *P *= 0.055) in cancer tissue compared with adjacent tissue. Therefore we believe that there is no confliction between the both reports. Further, serum FGF19 significantly decreased after curative hepatectomy. Therefore, FGF19 appears to function in an autocrine or paracrine fashion. *FGF19 *mRNA expression was correlated with prognostic significance for the survival of patients with HCC. Thus, this is the first report showing the clinical importance of FGF19. These *in vitro *studies revealed that recombinant FGF19 could induce proliferation, stimulate invasion, and inhibit apoptosis in HCC lines. Inversely, the introduction of *FGF19 *and *FGFR4 *siRNA was able to reduce proliferation and increase apoptosis in HCC lines. These findings suggest that the FGF19/FGFR4 system plays a critical role in HCC progression.

The mRNA expression levels of *FGF19 *and *FGFR4 *in HCC have been shown as 46% and 60%, respectively, by *in situ *hybridization [28]. In our study, all the examined human samples expressed both *FGF19 *and *FGFR4 *mRNA and the corresponding proteins. Further, all the cell lines examined expressed both *FGF19 *and *FGFR4 *at the higher level than normal hepatocyte. These results suggest that FGF19/FGFR4 are generally expressed in HCC. We investigated the correlation between *FGF19 *expression and the clinicopathological parameters in HCC. Our data indicated significant correlation in our cohort between *FGF19 *expression and pathological stage. The reason why *FGF19 *had no significant correlation with other prognostic factors, such as tumor size and vessel invasion, may be the small number of samples included in this study. Univariate and multivariate analyses revealed FGF19 to be an independent prognostic factor in HCC patients. These results indicate that high levels of FGF19 might help to identify HCC patients with poor prognoses and that FGF19 could be a novel prognostic marker of HCC. However, the detailed mechanism by which high levels of FGF19 contributes to the poor prognosis of patients with HCC must be elucidated by further study. However, we consider that FGF19 is certainly involved in the progression of HCC because recombinant protein and siRNA transfection were capable of affecting proliferation, invasion, and apoptosis in the experimental models.

Previous multivariate analyses have identified several variables as independent predictors of patient prognosis (tumor size, number of lesions, portal vein invasion, etc.) in patients who were not treated surgically and in those who underwent surgical resection. Further, a number of molecular markers with prognostic significance have been identified in HCC [32]. In this study, we showed that FGF19 is a novel independent prognostic factor for both disease-free and overall survival (Table 3). Moreover, serum FGF19 levels significantly decreased in HCC patients after curative operations (Figure 7). The sensitivity of serum FGF19 renders it a promising tumor marker for HCC. These results strongly indicate that FGF19 in surgically excised HCC tissues or preoperatively in the blood serum might help to identify patients with aggressive disease who will need adjuvant therapy.

In this study, we examined whether the introduction of siRNA into HCC cell lines could suppress the proliferative and anti-apoptotic properties of the HCC cell lines *in vitro. FGF19 *siRNA successfully suppressed *FGF19 *mRNA and FGF19 protein expression in the high FGF19-expressing cell line JHH7. Further, the proliferative and anti-apoptotic properties of JHH7 were lost *in vitro *after FGF19 siRNA transfection. Transfection of *FGFR4 *siRNA showed similar results. Recently, Desnoyers et al. have reported that neutralizing antibody of FGF19 treatment significantly suppressed the growth of established colon cancer tumors *in vivo *[28]. RNAi has been effectively used in target-directed therapies in a range of diseases and in the silencing of tumor genes by systemically administered siRNA *in vivo *[33-35]. Likewise, with antibody treatment, *FGF19 *and *FGFR4 *siRNA also may be potential targets for systemic therapy for HCC.

## Conclusions

In conclusion, our results demonstrated that FGF19 expression was significantly upregulated in HCCs, with higher expression being correlated with poor prognosis. Our results raise the possibility for potential treatment strategies aimed at functional abrogation of *FGF19 *that provide new therapeutic approaches in the management of HCC.

## Abbreviations

siRNA: Small interfering RNA; FGF: Fibroblast growth factor; HCC: Hepatocellular carcinoma; 5-FU: 5-fluorouracil; RT-PCR: Reverse-transcription polymerase chain reaction.

## Competing interests

The authors declare that they have no competing interests.

## Authors' contributions

SM participated in the study design, carried out the *in vivo *experiments and evaluation of immunohistochemical staining, and drafted the manuscript. NM participated in the study design and helped to draft the manuscript. HS and FK coordinated and performed the statistical analysis and helped to draft the manuscript. HY and MO participated in the study design. AK, TS, and DO carried out *in vitro *experiments and performed statistical analysis. MM participated in the study design, carried out evaluation and validation of immunohistochemical staining, and helped to draft the manuscript. All authors have read and approved the final manuscript.

## Pre-publication history

The pre-publication history for this paper can be accessed here:

http://www.biomedcentral.com/1471-2407/12/56/prepub
